# Ilececum: A Comprehensive Review

**DOI:** 10.1155/2019/1451835

**Published:** 2019-02-03

**Authors:** Shou-jiang Tang, Ruonan Wu

**Affiliations:** Division of Digestive Diseases, Department of Medicine, University of Mississippi Medical Center, USA

## Abstract

For gastrointestinal endoscopists, the ileocecum is the finishing line during colonoscopy and it is identified by three endoscopic landmarks: terminal ileum, ileocecal valve, and the appendiceal orifice. Although ileal intubation is recommended during routine screening colonoscopy, it is not required in most cases of screening colonoscopy. Ileal intubation is indicated in certain circumstances such as suspected inflammatory bowel disease and GI bleeding. There is much pathology that can be observed within the ileocecum. Careful and systematic examination should be stressed during GI endoscopic training and practice. In this review, the authors demonstrate its anatomy, endoscopic findings, and pathologies.

## 1. **Background**

For gastrointestinal (GI) endoscopists, the ileocecum is the finishing line during colonoscopy and it is identified by three endoscopic landmarks: terminal ileum (TI), ileocecal (IC) valve, and the appendiceal orifice. Although ileal intubation is recommended during routine screening colonoscopy, it is not required in most cases of screening colonoscopy. Ileal intubation is indicated in certain circumstances such as suspected inflammatory bowel disease (IBD) and GI bleeding. There is much pathology that can be observed within the ileocecum. Careful and systematic examination should be stressed during GI endoscopic training and practice. In this review, the authors demonstrate its anatomy, endoscopic findings, and pathologies. The complete one-hour digital video content pertaining to this review was published recently by the American Society for Gastrointestinal Endoscopy and is available at https://learn.asge.org/Public/Catalog/Details.aspx?id=vhdgXoOC8Uu9UNuyTKSTpw%3d%3d&returnurl=%2fUsers%2fUserOnlineCourse.aspx%3fLearningActivityID%3dvhdgXoOC8Uu9UNuyTKSTpw%253d%253d.

## 2. Anatomy, Imaging Study, and Endoscopic Examination

**Figure 1:**
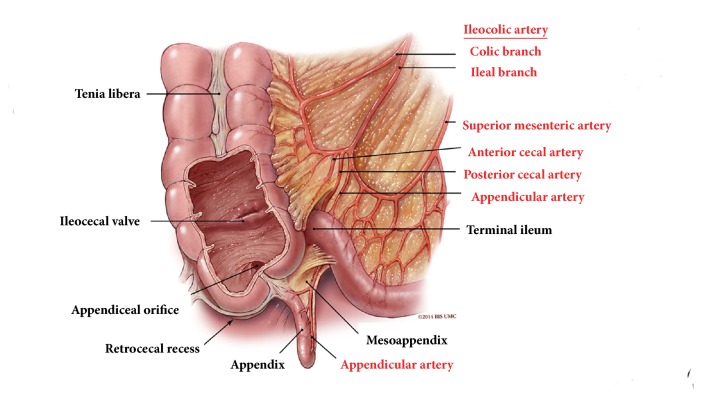
Illustration showing the anatomy and its vascular supply of the ileocecum: terminal ileum, ileocecal valve, cecum, and the appendix. The superior mesenteric artery (SMA) supplies the ileum, cecum, and the ascending colon. SMA branches into ileal arteries and forms anastomotic loops or arcades. From these arcades, the straight arteries supply the small and large bowels. The appendix is supplied by the appendicular artery which arises from the terminal branch of the SMA instead of the arterial arcades.

**Figure 2:**
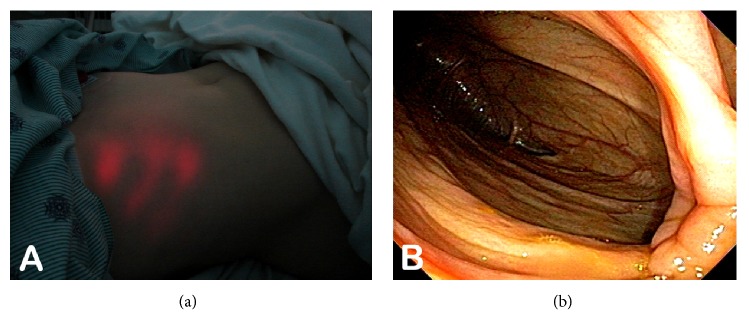
Images of a patient with gut malrotation, the cecum is located within the right upper quadrant, below the liver. Trans-abdominal light illumination can be observed within the right upper quadrant (a). The hepatic hue is observed during colonoscopy (b).

The cecum is a blind pouch of the colon ranging 6 cm-9 cm in length (Figure 1). The average length of the appendix is about 8 cm and is suspected from the TI by the mesoappendix [1, 2]. The appendiceal orifice is located about 2 cm-3 cm below the IC valve. In terms of cecal shape, there are generally four types: normal, exaggerated, conical, and quadrate shapes. The majority of the population has a normal type where the right saccule is larger than the left one. In the exaggerated type, the left saccule is atrophied and the appendiceal office is right next to the IC valve. In fetal or conical type, the cecum becomes conical in shape. In the quadrate or infantile type, the right and left saccules are identical in size. Depending on the variations in posterior peritoneal attachment of the cecum, the cecum can be either partially or completely intraperitoneal. The cecum is normally located within the right lower quadrant of the abdomen. In patients with congenital gut malrotation, the cecal location can vary (Figure 2). The superior mesenteric artery (SMA) supplies the ileocecum and ascending colon. SMA branches into ileal arteries and forms anastomotic loops or arcades. From these arcades, the straight arteries supply the small and large bowels. The appendix is supplied by the appendicular artery which arises from the terminal branch of the SMA instead of the arterial arcades. There is significant amount of gut associated lymphoid tissue in the ileocecum. The lymphatic drainage of the ileocecum follows the mesoappendix, ileocolic artery, and superior mesenteric lymph nodes [3]. The appendix has been hypothesized to be an immune organ and acts as a reservoir for normal gut flora [4, 5]. The IC sphincter has a sustained tone and provides a clearance mechanism for reflux of colonic contents into the small bowel [6]. The presence of short chain fatty acids in the TI is an important factor in triggering this clearance mechanism. The IC valve has been hypothesized to play a role in the pathophysiology of pain, bloating, and altered bowel movements in patients with irritable bowel syndrome [7].

Radiologists generally refer the ileocecum as the “ileocecal area” [8, 9]. Although the multidetector row computed tomography (CT) is currently considered the best imaging examination for the evaluation of the ileocecum, the diagnosis can occasionally be challenging [8–10]. On CT scan, the normal IC valve can have many different appearances, depending on cecal distention and mobility, whether the valve is open or closed, and inherent variable morphologic characteristics [8, 9]. In addition, flat cecal lesions are difficult to detect, and larger masses are sometimes mistaken for the IC valve or residual stool.

**Figure 3:**
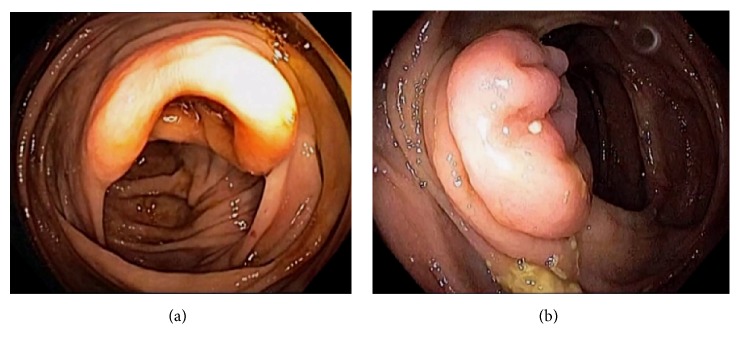
Endoscopic images of the ileocecal valve in labial (a) and papillary (b) forms.

**Figure 4:**
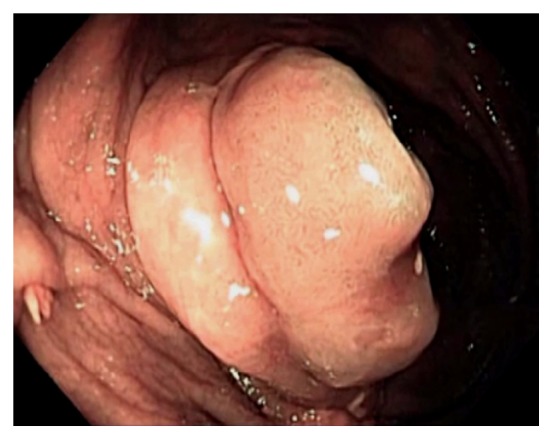
Endoscopic image of the ileocecal valve under digital chromoendoscopy. The small bowel mucosa protrudes or extends outside the ileal opening mimicking an adenoma.

**Figure 5:**
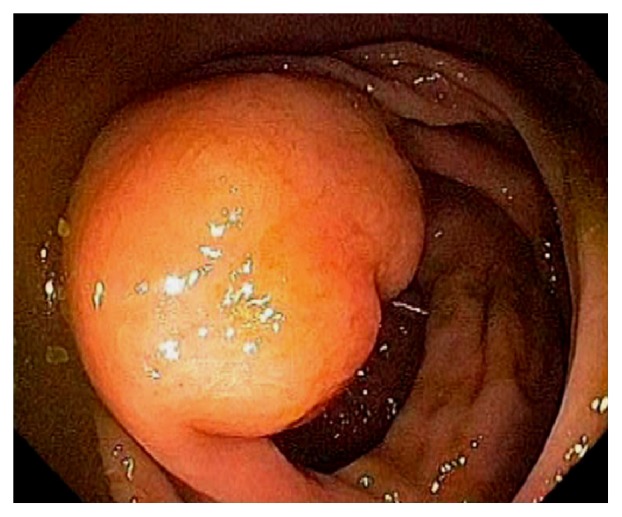
Endoscopic image of lipomatous hyperplasia of the ileocecal valve due to focal proliferation of adipose tissue within the submucosa of the valve.

Endoscopically, the IC valve can appear as either labial or papillary form (Figure 3). In most cases, the labial type valve is seen during colonoscopy. The vascular patterns and mucosal pathology can be better visualized and demarcated under digital chromoendoscopy, such as narrow band imaging. The normal mucosal lining of the valve is colonic epithelium on the outside and small bowel mucosa inside. Sometimes, the small bowel mucosa can protrude or extend outside the ileal opening mimicking an adenoma (Figure 4). In this scenario, the villous pattern of the mucosa can be traced back into the TI. Submucosal injection of a contrast agent (submucosal chromoendoscopy) and digital chromoendoscopy can usually highlight the dysplastic mucosa if present. Whenever in doubt, endoscopic biopsy is recommended for confirmation. Not infrequently, lipomatous hyperplasia of the IC valve can be observed (Figure 5). This is a benign condition due to focal proliferation of adipose tissue within the submucosa of the valve.

**Figure 6:**
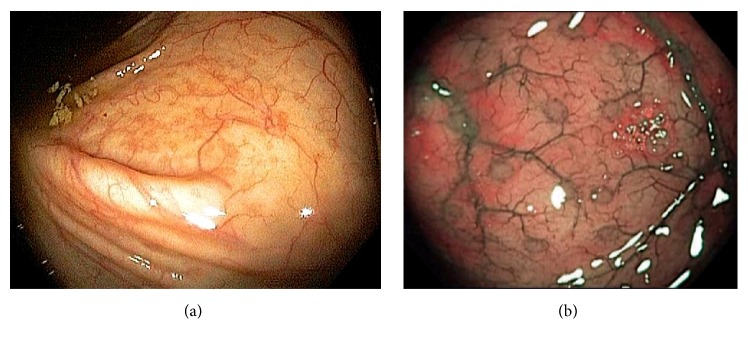
Endoscopic images of the cecum. Normal vascular pattern within the ileocecum consists of branching vascular network within background of pink mucosa and intramural arteries penetrating the colonic wall (a). Gut associated lymphoid tissue is abundant in the ileocecum and they can appear as Peyer's patches, lymphoid nodules, and periappendiceal and appendiceal lymphoid follicles. These normal findings are best observed under digital chromoendoscopy (b).

**Figure 7:**
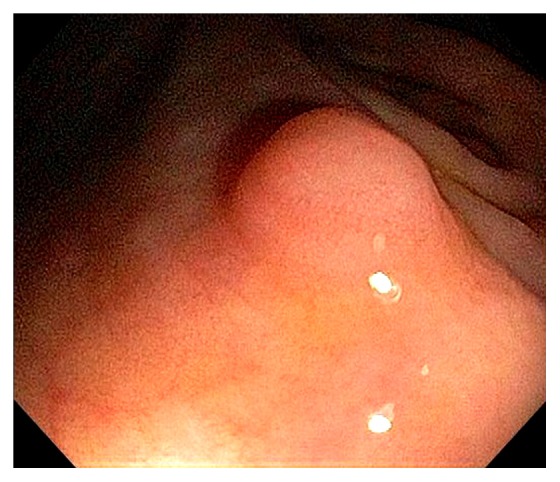
Endoscopic image of a reactive lymphoid polyp in the cecum.

Normal vascular pattern within the ileocecum consists of a branching vascular network within the background of pink mucosa and intramural arteries penetrating the colonic wall. The vascular pattern is less prominent at the IC valve. Sometimes, fine vascular pattern can also be observed within the TI. Gut associated lymphoid tissue is abundant in the ileocecum, and they can appear as Peyer's patches, lymphoid nodules, periappendiceal and appendiceal lymphoid follicles (Figure 6). Bowel preparation can “highlight” these lymphoid tissue and causes patchy erythema, erosions or aphthoid ulcers, increased lymphocytes and neutrophils in the mucosa [11–13]. Within the TI, the lymphoid follicles can occasionally appear as tiny pedunculated polyps. Infrequently, reactive lymphoid polyp can be found in the cecum (Figure 7). When indicated or in doubt, endoscopic biopsy should be performed to rule out submucosal neoplasms, such as carcinoids. The cecum has the thinnest wall thickness of the entire colon, ranging between 2 mm - 3 mm. The pelvic vasculature may appears as bluish discoloration seen through the cecal wall. The appendiceal orifice lies at the junction of three tenia coli at the cecal pole. This has been referred to as the “craw's foot” appearance of the cecum. During colonoscopy, the tenia coli are often indistinctive, and the classic “craw's foot” appearance of the cecum is absent. Occasionally, a cecal diverticulum can mimic the appendiceal orifice and vice versa. After appendectomy, the appendiceal orifice may appear as a small dimple at the cecal pole. A partially invaginated appendiceal stump can be noticed as a protuberance at the orifice in patients after appendectomy.

**Figure 8:**
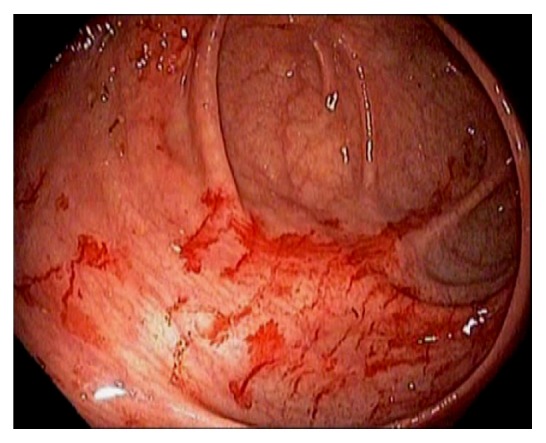
Endoscopic image of mucosal findings of barotrauma in the cecum.

**Figure 9:**
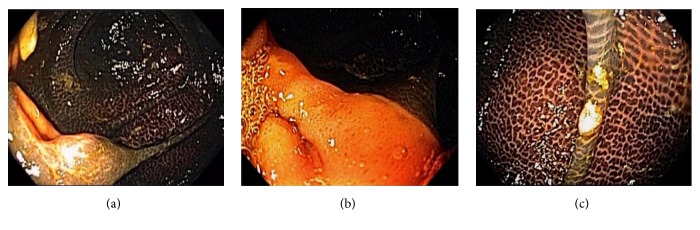
Endoscopic images of pseudomelanosis coli. The small bowel mucosa inside the terminal ileum and at the ileocecal opening is spared from pigmentation (a). Spotty pigmentation at the ileocecal opening can be observed within the transitional zone (b). Colon adenomas usually lack pigmentation and are highlighted by pseudomelanosis (c).

**Figure 10:**
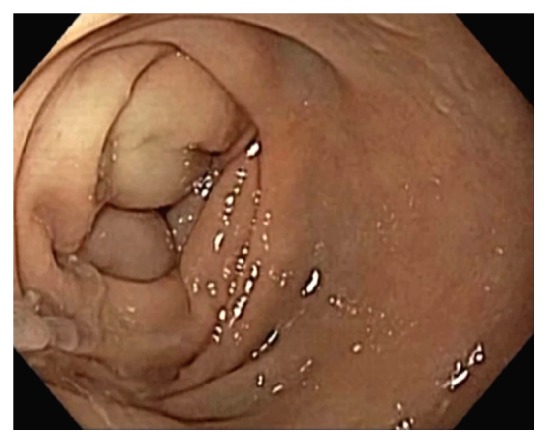
Endoscopic image showing generalized gut edema in a patient with severe hypoalbuminemia. The ileocecal wall appears edematous with a loss of mucosal vascular patterns.

TI intubation is considered to be the gold standard in successful cecal intubation, followed by identification of the IC valve and appendiceal orifice [14, 15]. Various techniques can be used to intubate the IC valve and terminal ileum, such as direct visualization, scope retroflexion in the cecum, using a closed biopsy forceps as a lead “guide wire” under “low air conditions”, etc. Identification of the appendiceal orifice alone or trans-illumination through the abdominal wall can be falsely positive in more than 10% of colonoscopies [14]. The authors recommend that endoscopic examination of the ileocecum should start from the appendiceal orifice, cecal examination in circular fashion or circumferentially, examination of the IC valve on face and behind the proximal valve, and TI intubation. Optimal visualization of the ileocecum can be achieved by torqueing the endoscope with the right hand and gently maneuvering the wheels on the handle using the left hand. Some experts advocate performing retroflexion of the endoscope in the cecum and ascending colon. This endoscopic maneuver provides an optimal view of the valve and the cecal area behind the valve. In addition, retroflexion of the endoscope can be used to achieve ileal intubation in difficult cases and for polypectomy if the lesion is situated proximal to the valve. Because the cecum is at the dependent location, the colon preparation and endoscopic visualization can be suboptimal. If only liquid stool is seen in the cecum, aggressive lavage should be performed to achieve optimal visualization of the cecum. Not infrequently, we can observe barotrauma within the cecum and the proximal ascending colon (Figure 8). This is from submucosal hemorrhage due to air insufflation. The likely mechanism is air over insufflation while the patient is in left lateral position. The mucosal findings in barotrauma can be linear or patchy and can mimic cecal angioectasia. After chronic use of anthranoid containing laxatives or food, the colon mucosa may appear as brown, i.e., pseudomelanosis coli [11, 12] (Figure 9). The brownish pigmentation is a benign condition and is caused by the accumulation of lipofuscin within the apoptotic epithelial cells [11, 12]. It can develop after several months of anthranoid usage. Small bowel mucosa inside the TI and at the IC opening is spared from pigmentation. Colon adenoma usually lack pigmentation and are highlighted by pseudomelanosis. Generalized gut edema can be observed in patients with severe hypoalbuminemia and portal hypertension (Figure 10). Endoscopically, the cecal wall appears edematous with a loss of mucosal vascular patterns.

## 3. Cecal Dilation

**Figure 11:**
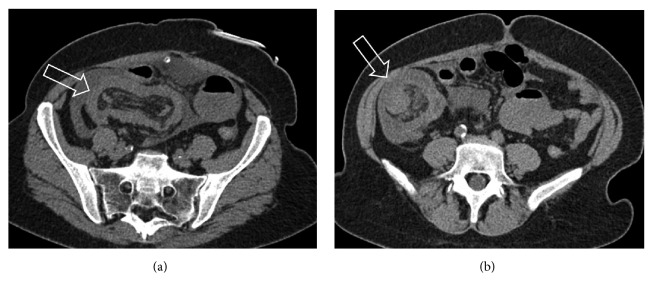
CT scan images showing ileocecal intussusception (arrows) from a 2 cm neuroendocrine tumor arising from the terminal ileum.

On imaging studies, the upper limit of normal cecal diameter is < 9 cm. Cecal dilation >9-12 cm can cause ischemia and necrosis. A variety of obstructive and nonobstructive etiologies can cause cecal dilation, such as cecal volvulus, ileocecal intussusception, distal colon obstruction, colon inertia, acute colonic pseudoobstruction, infections, and ischemia. Megacolon can be caused by Clostridium difficile colitis, cytomegalovirus infection, and severe ulcerative colitis (UC). CT findings in cecal volvulus include cecal distention (>10 cm), coffee bean sign, abnormal cecal apex location (left upper quadrant), whirl, ileocecal twist, transition point(s), and distal colon decompression [16]. Unlike sigmoid volvulus, colonoscopy is generally not useful and indicated in cecal volvulus or surgery is required [17]. Surgery consult is required in intussusception for the risk of ischemia and the possibility of a malignant lead point [18, 19] (Figure 11). The length and diameter of the intussusception, presence of a lead point, or bowel obstruction on CT scan are predictive of findings that warrant surgical exploration [18]. The lead point can be a neoplasm, infection, ischemia, or enterocolic lymphocytic phlebitis [19, 20]. Benign IC valve hypertrophy, also called Bauhin's IC valve syndrome, is a rare cause for small bowel obstruction [21].

## 4. Diverticulum and Inverted Appendix

**Figure 12:**
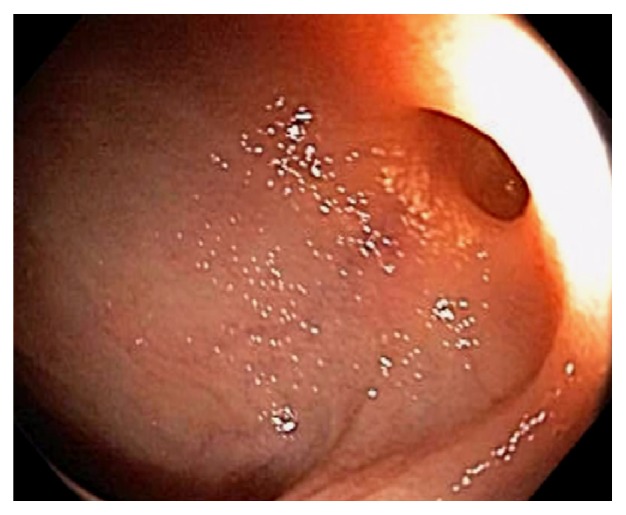
Endoscopic image of an uncommon ileal diverticulum (right upper corner) in the ileocecum.

**Figure 13:**
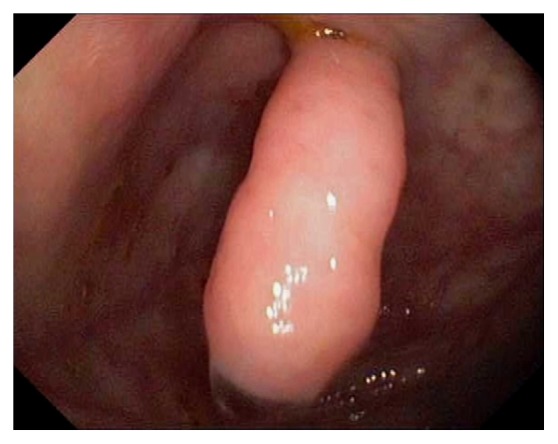
Endoscopic image of an inverted appendix appearing as a polypoid lesion at the cecal pole. The mucosal pattern is normal.

Occasionally, we can observe diverticulosis in the ileocecum, either as a single diverticulum or as multiple diverticula (Figure 12). Infrequently, a fecalith can be seen at the diverticular opening. A small percentage of patients with diverticulosis can develop diverticulitis and diverticular bleeding [22, 23]. Ascending colon and cecal diverticulum can mimic the appendiceal orifice, leading to incorrect call of cecal intubation and missed lesions. Rarely, the appendix and diverticulum can be inverted, mimicking a polyp (Figure 13). Inverted appendix is also called appendiceal intussusception [24–26]. Appendiceal intussusception can result from no obvious underlying pathology as an incidental finding, a fecalith, inflammation, endometriosis, mucocele, or appendiceal neoplasms. Appendiceal intussusception from certain pathologies, such as neoplasm, can evolve into ileocecal intussusception and bowel obstruction. There have reported cases of perforation after “polypectomy” of inverted appendix [27–29]. After unintentional polypectomy, if the endoscopist realizes the possibility of inverted appendix, it is prudent to apply endoscopic clipping devices to close the resection margins.

## 5. Hemorrhage

**Figure 14:**
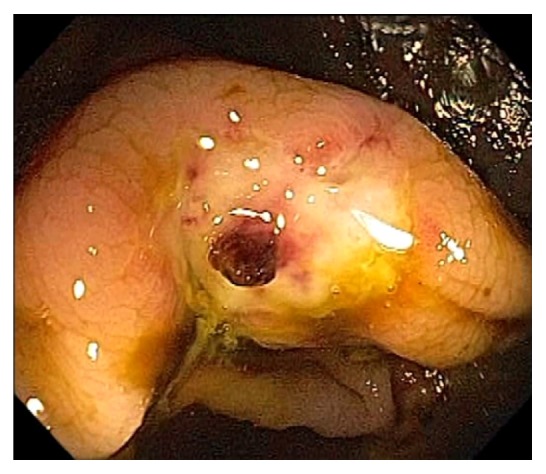
Endoscopic image of postpolypectomy cecal ulcer with a visible vessel.

**Figure 15:**
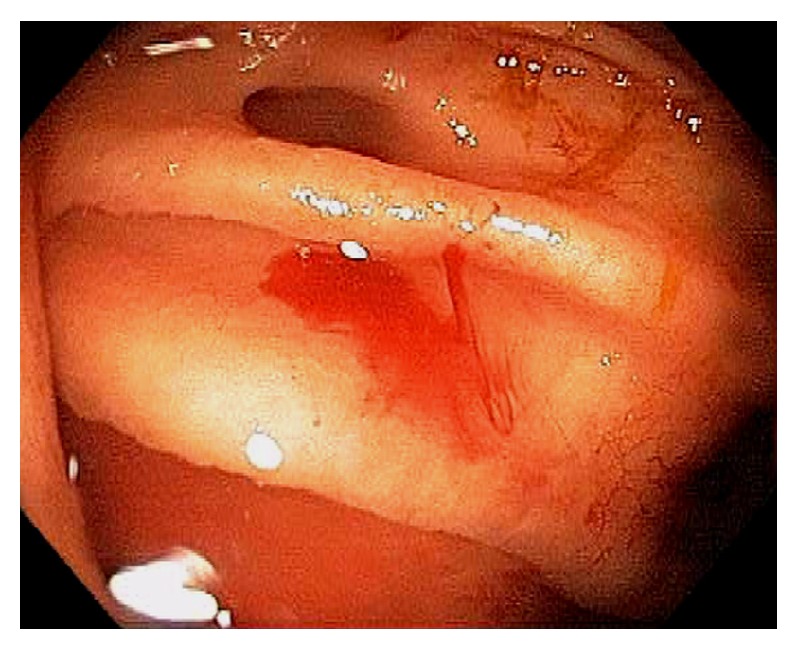
Endoscopic image of a bleeding angioectasia. Fresh blood oozes from a punctate spot.

In the diagnosis of GI bleeding, it is important to recognize whether the bleeding source is located proximal or distal to the IC valve. During endoscopy, the absence of blood in the TI while the colon has fresh blood significantly reduces the possibility of proximal GI bleeding. However, during severe GI bleeding from a colonic source, a small amount of blood can reflux into the TI. It is important to recognize that food coloring or color additives can mimic blood, and certain medications, such as iron supplements, activated charcoals, and bismuth can turn stool black. Common etiologies for ileocecal bleeding include vascular lesions, such as angioectasia, Dieulafoy's lesion, varices, diverticular bleeding, ulceration and erosion, postpolypectomy bleeding (Figure 14), fistula, endometriosis, and neoplasm. Cecal angioectasia can be an incidental finding, and it can also cause chronic GI blood loss leading iron deficiency anemia. In a patient who is on anticoagulation therapy, overt GI bleeding, such as melena, can develop. During colonoscopy, air over insulation or flushing with room temperature water can cause blanching of the angioectasia or masking of the lesion. If actively bleeding, we generally do not see a spider shaped angioectasia. Fresh blood can be seen oozing from a punctate spot where the angioectasia is located (Figure 15). Hemostasis can be achieved by applying thermal coagulation at the bleeding spot. Infrequently, we can observe active diverticular bleeding and this can be managed by injection therapy, followed by endoclip application and/or bipolar thermal coagulation. Bleeding from an appendiceal stump is a rare but severe complication after appendectomy [30]. The bleeding may occur into the abdominal cavity, the retroperitoneum, or the ileocecum and develop years after appendectomy [30]. If found during colonoscopy, endoscopic clipping for the treatment of appendiceal stump bleeding can be attempted [30].

## 6. Ischemia

**Figure 16:**
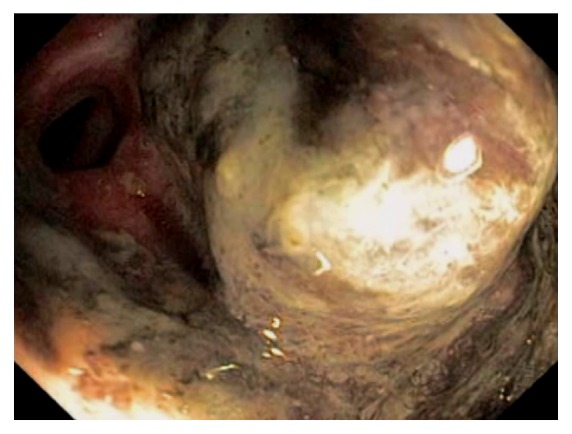
Endoscopic image of ischemic colitis involving the ileocecum. The appendix and appendiceal orifice are marginally spared and appear relatively normal.

Ischemic colitis or colon ischemia is generally induced by a low flow state, i.e., nonocclusive ischemia. Certain medications can induce colon ischemia, (1) directly through vasoconstriction (ergotamine and cocaine), (2) systemically through volume depletion (diuretics), or (3) regionally through mesenteric vein thrombosis related to the hypercoagulable status (estrogen supplementations) [11, 12, 31]. Ischemic colitis is usually left sided, affecting the sigmoid colon and splenic flexure. The ileocecum and ascending colon can be involved as well, either independently or in combination with left sided ischemic colitis. Cecal ischemia, infarction, necrosis, or gangrene have been used to describe ischemia of the ileocecum. On endoscopy, the mucosa appears blotchy or mottled and edematous. The normal mucosal vascular pattern is absent. Erosion and ulceration can be present. Biopsy is recommended to confirm the diagnosis and to rule out other etiologies of ulceration. Even in severe ischemic colitis involving the ileocecum and ascending colon, the TI is relatively spared in the ischemic process, likely due to decreased wall tension and relatively richer vascular network in the small bowel. Due to its separate blood supply, the appendix and appendiceal orifice are marginally spared and appear relatively normal as well [32] (Figure 16). The patients with ischemic colitis can present with gross GI bleeding, right lower abdominal quadrant pain, fever, and leukocytosis. CT scan usually reveals circumferential wall thickening of the cecum, occasional pneumatosis coli and areas of low and high attenuations representing intramural edema and hemorrhage. In severe ischemic colitis, bacterial superinfection is a potential cofactor in the pathogenesis [32]. The management of right-sided ischemic colitis is generally supportive.

## 7. Drug-Induced Injury

**Figure 17:**
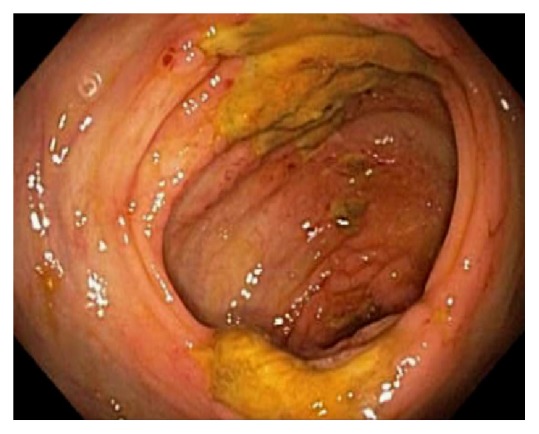
Endoscopic image of medication induced mucosal injury in the ileocecum, on the valve and the opposite of the valve. In this case, medication crystals are found on biopsy specimen.

A variety of medications can cause injury to the ileocecum, either directly or indirectly [11, 12, 33–35]: nonsteroidal anti-inflammatory drugs (NSAIDs), alendronate, mycophenolate mofetil, monoclonal antibodies, sodium polystyrene sulfonate, bowel preparation, etc. Medication induced injury includes villous atrophy, cryptitis, crypt distortion, apoptosis, and ulceration [11, 12, 33]. Occasionally, pathologists can obverse medication crystals on biopsy specimen, such as with sodium polystyrene sulphonate and sevelamer [12]. They can mimic inflammatory bowel disease, microscopic colitis, eosinophilic colitis, ischemic colitis, and graft versus host disease [11, 33]. On endoscopy, patchy mucosal edema, erythema, erosions, ulcerations, stricture in the TI, and colonic diaphragm can be observed (Figure 17). Endoscopic biopsy should be performed to rule out other causes such as infection.

**Figure 18:**
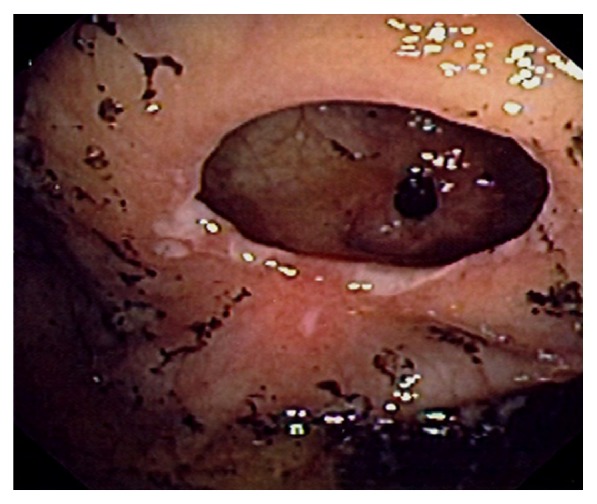
Endoscopic image of cecal diaphragm disease due to nonsteroidal anti-inflammatory drugs.

NSAIDs are known to cause erosion and ulceration in the ileocecum. Pathological findings on biopsy are nonspecific [11]. Diffuse circumferential ulceration can lead to stricture or diaphragm formation [11, 12, 33, 34], usually induced by sustained-release NSAIDs. The mucosa can appear normal between multiple web-like fibrotic constrictions or diaphragms [34] (Figure 18). Besides cessation of the NSAIDs, in symptomatic patients with diaphragm disease, endoscopic dilation can be performed, such as using 15 mm - 20 mm size balloons for colon diaphragms [33, 34]. Sodium phosphate in certain colon preparations can cause aphthous-like lesions, appearing as small foci of pale mucosa surrounded by erythematous rings that mimic shallow erosions [11]. Histologically, they are large lymphoid aggregates. Infrequently, oral sodium phosphate can induce patchy neutrophilic cryptitis or focal active colitis [11].

## 8. Foreign Body Impaction

**Figure 19:**
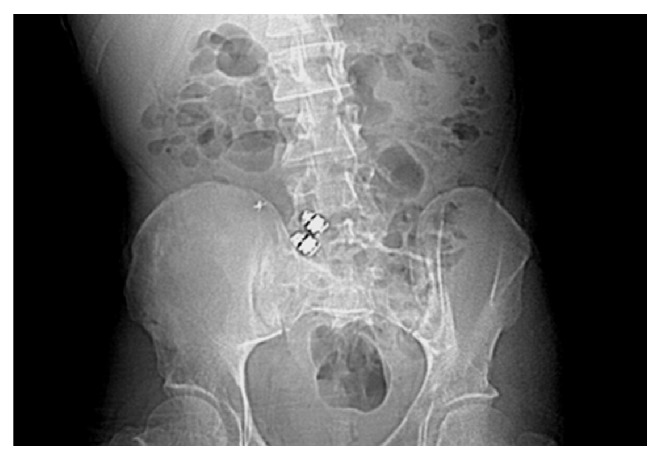
Radiological image of foreign body (capsule endoscopes) impaction can develop if stricture is present in the ileocecum or ascending colon.

**Figure 20:**
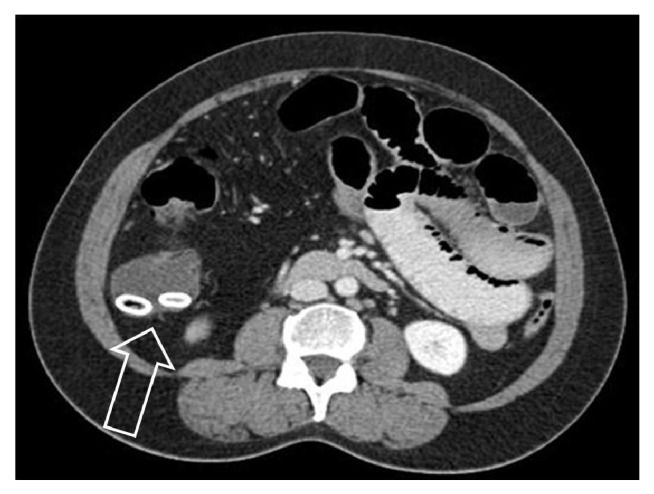
Computer tomographic image of two calcified fecaliths (arrow) in the cecum in a patient with ascending colon and ileal strictures due to Crohn's disease.

Once entered the small bowel, the swallowed foreign bodies should pass through the ileocecum. Serial X-ray studies are recommended to ensure the safe passage of these foreign bodies. Foreign body impaction can develop if the patient has stricture in the ileocecum (Figure 19), or if the foreign body is of larger size such as a large gallstone. In addition, a sharp or pointed foreign body can penetrate the ileocecum [36, 37]. Due it its dependent location, pouch shaped cecum, and appendix, foreign body can reside in the ileocecum, sometimes leading to inflammation, perforation, and symptoms without downstream obstruction. A fecalith, also called fecolith, fecaloma, stercolith, and coprolith, refers to hardened fecal materials that can be calcified and stone-like. It develops in settings of chronic downstream obstruction or dysmotility. Cecum and appendix are common locations in the GI tract for fecaliths (Figure 20).

## 9. Fistulas

**Figure 21:**
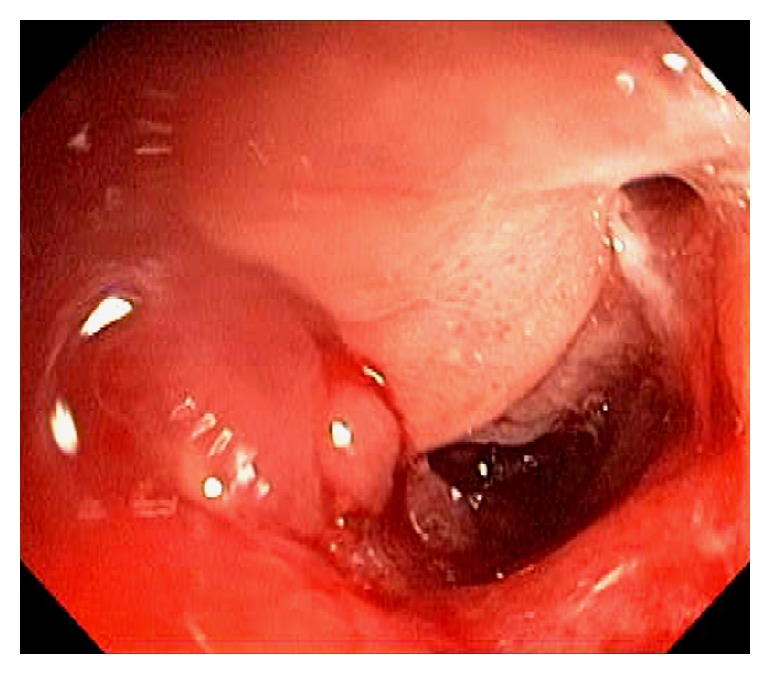
Endoscopic image of the ileocecal opening showing Crohn's ileitis with a fistula opening at 2 o'clock in the right upper corner.

Fistulas and sinuses are relatively common in the ileocecum from certain inflammatory conditions and infections, such as Crohn's disease, appendicitis, and tuberculosis (Figure 21). In addition, neoplasm of ileocecum, endometriosis, and right-sided ovarian pathologies can potentially lead to internal fistulization or entero-, ceco-, or appendicocutaneous fistulas. When local expertise is available, endoscopic therapy can be attempted, such as clipping, suturing, local tissue adhesive application [38, 39].

## 10. Portal Colopathy and Varices

**Figure 22:**
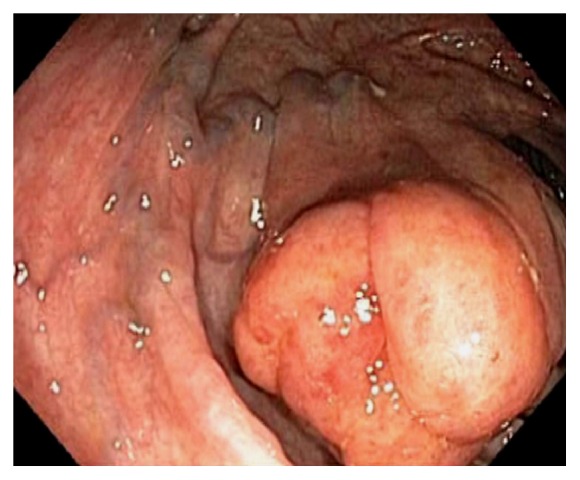
Endoscopic image of ileocecum showing mucosal edema, mild loss of mucosal vascular pattern, patchy erythema, vascular ectasia, and varices. The ileocecal valve is edematous in the left lower corner.

Both small and large bowels are involved in portal hypertension or mesenteric thrombosis, leading to portal hypertensive enteropathy and colopathy [40–42]. Endoscopic findings within the ileocecum include mucosal edema, loss of mucosal vascular pattern, patchy erythema, vascular ectasia, and varices (Figure 22). The IC valve is generally edematous.

## 11. Inflammation and Infection

**Figure 23:**
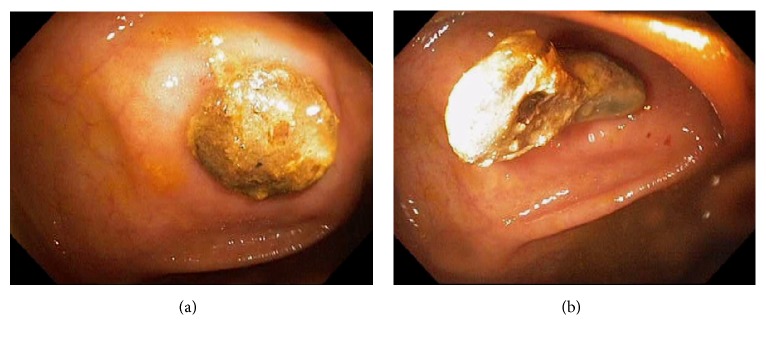
Endoscopic images of a fecalith impacting the appendiceal drainage (a) in a patient with recurrent right lower abdominal pain. Endoscopic disimpaction of fecalith can be attempted (b).

**Figure 24:**
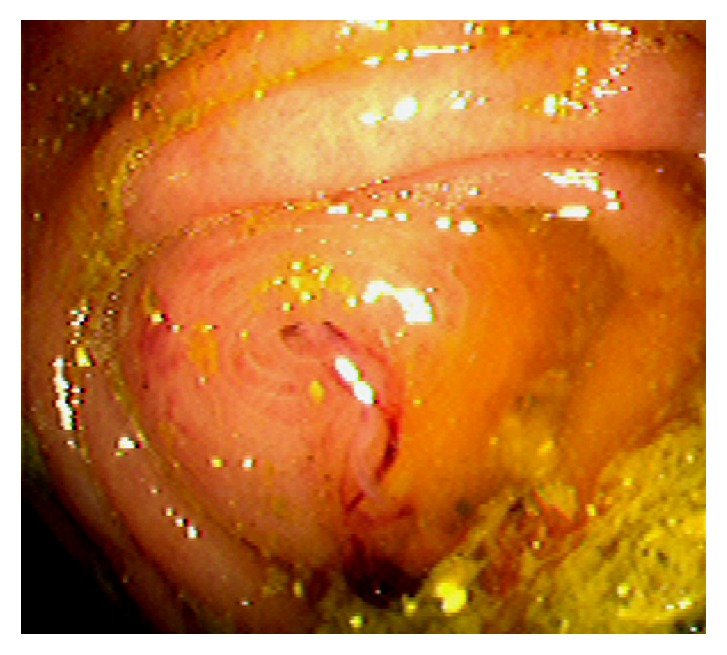
Endoscopic image of acute appendicitis.

Acute appendicitis is a common differential diagnosis when patients present with right lower abdominal pain and other GI symptoms [43, 44]. Impaired appendiceal drainage with subsequent inflammation can be caused by fecalith, mucocele, neoplasm, and endometriosis (Figure 23). The lifetime risk for appendicitis is about 6% - 9% [45]. Infrequently, acute appendicitis can develop after colonoscopy and partially resected appendix, i.e., stump appendicitis [46–49]. Colonoscopy may be useful in the diagnosis of appendicitis when the clinical presentation is atypical for appendicitis and/or imaging studies are nondiagnostic [50, 51]. Endoscopic findings include a periappendiceal bulge, draining exudates from the appendiceal orifice, hyperemia and bulging at the appendiceal orifice area with surrounding mucosal edema, and drainage of pus from the appendiceal orifice (Figure 24). Occasionally, endoscopist can find the inducing pathology such as a blocking fecalith. Complications of appendicitis include perforation, abscess formation, and peritonitis. CT scan is a highly accurate and noninvasive test for appendicitis [52]. The standard treatment options include antibiotics and surgical appendectomy. Recently, there is evidence to support the management of patients with uncomplicated acute appendicitis with antibiotics alone [43, 53]. In addition, there are early reports of endoscopic retrograde appendicitis therapy and endoscopic retrograde appendicography in patients with uncomplicated appendicitis [54–56]. Besides noninvasive CT scan and trans-abdominal ultrasound examination of the appendix, contrast study of the appendix has also been reported through enema, endoscopic retrograde appendicography, and direct appendicoscopy [57–59]. Acute epiploic appendagitis is a rare and often misdiagnosed cause of acute abdominal pain. Though a benign and often self-limiting condition, epiploic appendagitis often mimics other disease processes making it an important consideration in patients presenting with acute abdominal symptoms [60]. Careful evaluation of abdominal CT scan findings is crucial in the accurate diagnosis of epiploic appendagitis.

**Figure 25:**
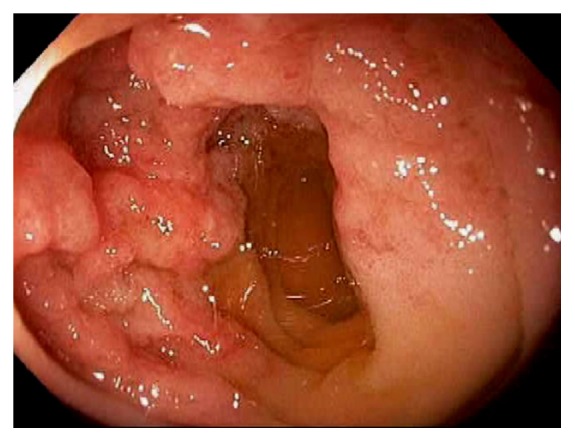
Endoscopic image of the terminal ileum during acute infectious ileocolitis. The mucosa is edematous, erythematous, and with prominent lymphoid tissues.

**Figure 26:**
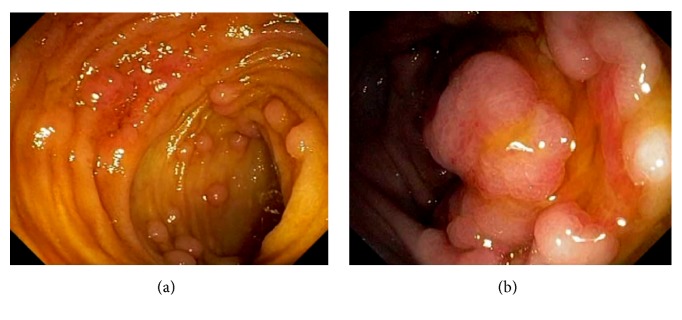
Endoscopic image of ileocecal histoplasmosis: terminal ileum (a) and cecum (b).

Due to its rich gut associated lymphoid tissue, many infections tend to involve the ileocecum, such as tuberculosis. Although any area of the gut can be involved in tuberculosis, the common site of the GI tract is the ileocecum [61–63]. Endoscopic appearance of diseased mucosa can be nonspecific. Radiological findings of abdominal tuberculosis can mimic those of many different diseases. Adequate bacteriological and histological assessment of biopsied tissue is essential to differentiate tuberculosis from other disorders. GI and peritoneal tuberculosis are treated with antituberculous drugs. Surgery is reserved for complications or uncertainty in diagnosis. Infectious ileocecitis caused by Yersinia, Campylobacter, and Salmonella is a common mimicker of appendicitis [64] (Figure 25). In patients with typhoid fever, the most commonly involved area was the ileocecum and right-sided colon [65]. The most common endoscopic findings are punched-out ulcers with slightly elevated margin, hyperemic mucosal patches, hemorrhagic spots, or shallow erosions. Clostridium difficile causes the majority of cases of pseudomembranous colitis. Although the infection is pancolitis, its endoscopic presentation can be segmental and limited to cecum and ascending colon [66]. Actinomycosis is an uncommon chronic infectious disease. Common sites of involvement include the cervicofacial, thoracic and abdominopelvic regions. In abdominopelvic actinomycosis, ileocecum is the most commonly involved site [67]. Other infectious etiologies affecting the ileocecum include adenovirus, rotavirus, cytomegalovirus, amebiasis, histoplasmosis (Figure 26), crytosporidiosis, schistosomiasis, spirochetosis, strongyloides, syphilis, trichuriasis, and vibrio cholera.

**Figure 27:**
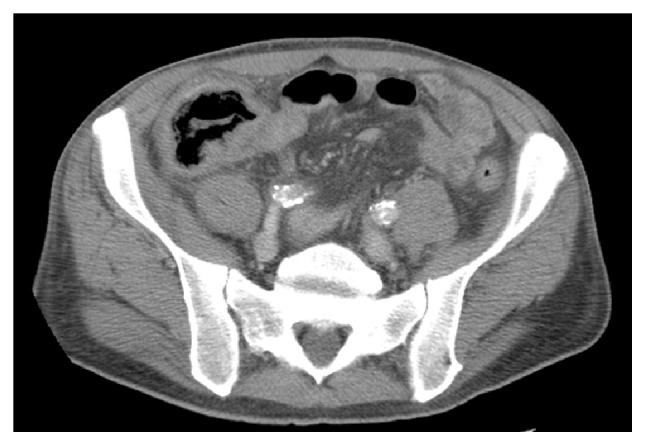
Computer tomographic image of typhlitis showing wall thickening involving the ileocecum and ascending colon, colon distention, and pneumatosis coli.

**Figure 28:**
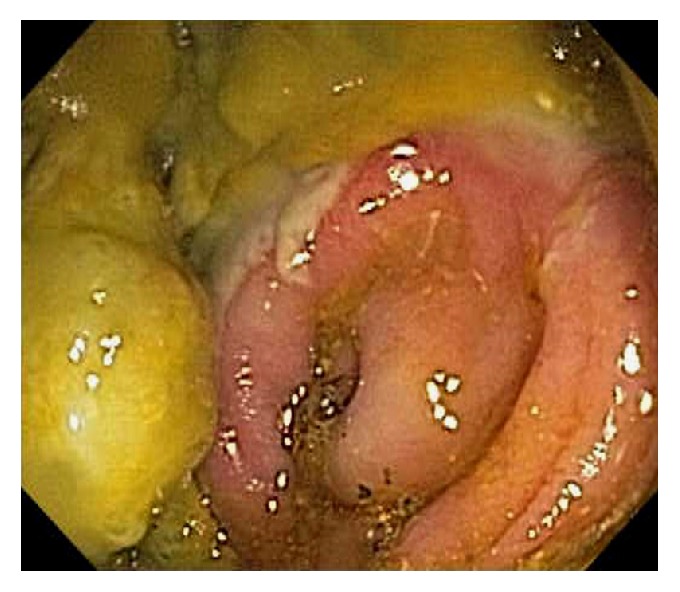
Endoscopic image of cecum in typhlitis. The appendix and periappendiceal mucosa are spared from ischemia and ulceration. Pneumatosis coli is seen on the left.

Neutropenic enterocolitis or typhlenteritis or typhlitis (from the Greek “typhlon” for cecum) is a life-threatening condition in patients with severe neutropenia after receiving chemotherapy and immunosuppression. The pathogenesis is likely from a combination of bowel wall ischemia, necrosis, bacterial translocation and superinfection [68–70]. The clinical presentation can be nonspecific due to coexisting neutropenia: abdominal pain, fever, diarrhea, ileus, and GI bleeding. The diagnosis is based on clinical presentation in neutropenic patients and CT scan. Typhlitis is a medical emergency and early diagnosis is paramount. On CT scan, wall thickening involving the ileocecum and ascending colon, colon distention, and pneumotosis coli can be present (Figure 27). Endoscopy should not be used as a diagnostic tool unless there are other luminal indications (Figure 28). The management of typhlitis is broad spectrum antibiotics, improving neutropenia, and supportive care. Surgery is reserved for intraperitoneal free air and perforation.

**Figure 29:**
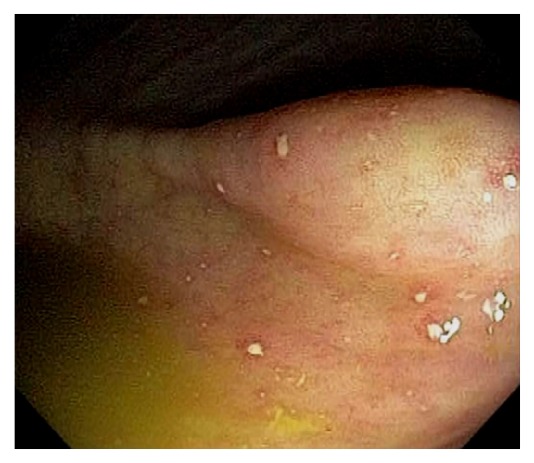
Endoscopic image of eosinophilic colitis showing patchy erythema with erosions.

**Figure 30:**
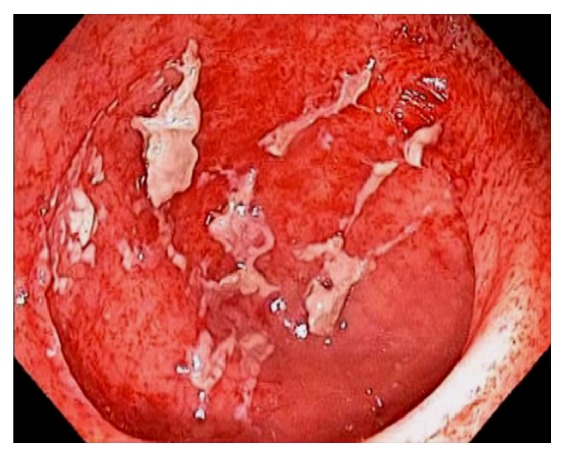
Endoscopic image of diversion colitis involving the cecum showing mucosal edema, erythema, friability, erosions, exudates, and barotrauma.

A variety of etiology can cause ileocecal inflammation such as Behcet's disease, colon diversion, eosinophilic enterocolitis (Figure 29), microscopic colitis, graft versus host disease, drug-induced injury, ischemia, radiation, and vasculitis. Diversion colitis involves nonspecific colonic inflammation following surgical diversion of the fecal stream away from the upstream colon [71]. Histopathologically, diffuse inflammation, crypt abscesses or atrophy, and lymphoid follicular hyperplasia can be observed. Endoscopically, mucosal edema, erythema, friability, erosions or ulcerations, exudates, and mucosal nodularities can be observed (Figure 30). Although most patients are asymptomatic, endoscopic evidence of diversion colitis can be found in the majority of patients after diversion. Restoring the fecal stream to the affected colon segment reverses diversion colitis. Behcet's disease is a chronic relapsing vasculitis of unknown origin that affects nearly all organs and systems [72–75]. The most frequent extra-oral sites of GI involvement are the ileocecal region and the colon. In Behcet's disease, ulcerations are frequently found in the ileocecum, large, deep, and prone to perforate [72, 75].

## 12. Inflammatory Bowel Disease

**Figure 31:**
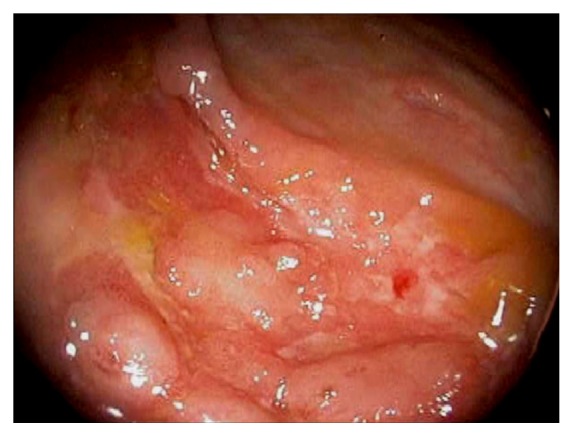
Endoscopic image of the cecum in a patient with Crohn's colitis. The ulcerations are generally large, deep, serpiginous, or geographic in morphology.

The ileum is a common organ to be involved in Crohn's disease. In patients with Crohn's ileitis, we can observe mucosal edema, erythema, erosions, ulcerations, stricture, scar formation, and fistula opening (Figure 21). Crohn's ulceration is generally large, deep, serpiginous, or geographic in morphology (Figure 31). Having slip lesions is another feature of Crohn's disease. The IC valve can be involved in Crohn's disease leading to ulceration, scar, and stricture formation. Endoscopic TI intubation can be challenging or impossible in such scenario. In symptomatic patients with TI stenosis at the IC valve, endoscopic balloon dilation can be attempted to relieve the symptom. In general, patients with fibrostenotic stricture, instead of with inflammatory stenosis, are more likely to respond to endoscopic therapy. Endoscopic biopsy is recommended to rule out an infectious cause and other etiologies. Occasionally, the TI can appear normal in patients with Crohn's colitis. It is important to obtain tissue diagnosis in situations where radiographic findings are consistent but atypical with the clinical diagnosis of Crohn's disease, such as mucinous adenocarcinoma of the ileocecal valve [76].

**Figure 32:**
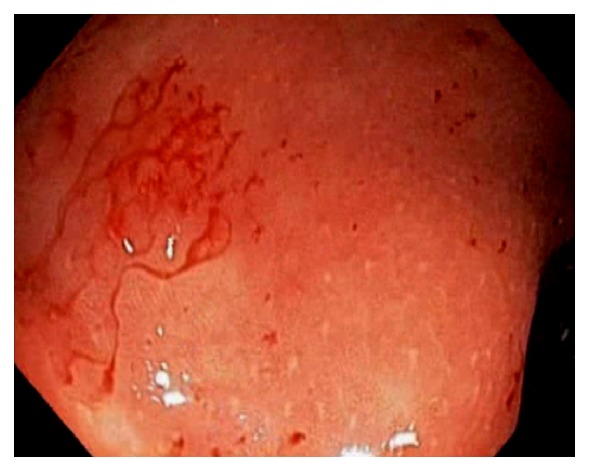
Endoscopic image of the cecum in a patient with ulcerative colitis. The colon mucosa is diffusely and continuously inflamed with small erosions or ulcerations, and petechial hemorrhage.

**Figure 33:**
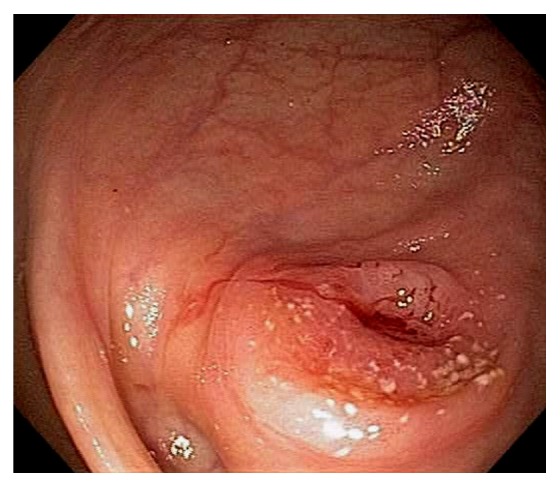
Endoscopic image of the cecum with “periappendiceal red patch” or “cecal patch”.

In patients with untreated UC, the colon mucosa is diffusely and continuously inflamed with small erosions, or ulcerations, and petechial hemorrhage (Figure 32). In patient with left sided UC, infrequently we can observe appendiceal orifice inflammation or “periappendiceal red patch” or “cecal patch” (Figure 33) [77–79]. The appendix has been hypothesized to play a role in mucosal immune function in the pathogenesis of UC [77–79]. The cecal patch appears as mucosal erythema, granularity, erosion, or ulceration. On biopsy, colitis findings can be seen. In one large cohort, the prevalence of cecal patch was 7.9% and all patients had appendix unremoved [79]. The clinical significance of “cecal patch” is unknown and it is has been considered to be a distinct “skip lesion” of ulcerative colitis and is frequently associated with distal, mild UC than extensive or severe disease [77–79]. Although it is still controversial, the cecal patch seems to have little prognostic implication in the disease course of UC, including remission, relapse and proximal disease extension [77]. Some preliminary data suggest that the appendix may have a role in the development of inflammatory bowel disease [80]. Appendectomy can potentially prevent or significantly ameliorate inflammatory bowel diseases in later life. The appendix may be linked to numerous immunological functions by acting as a reservoir for commensal gut flora [80].

## 13. Neoplasms

**Figure 34:**
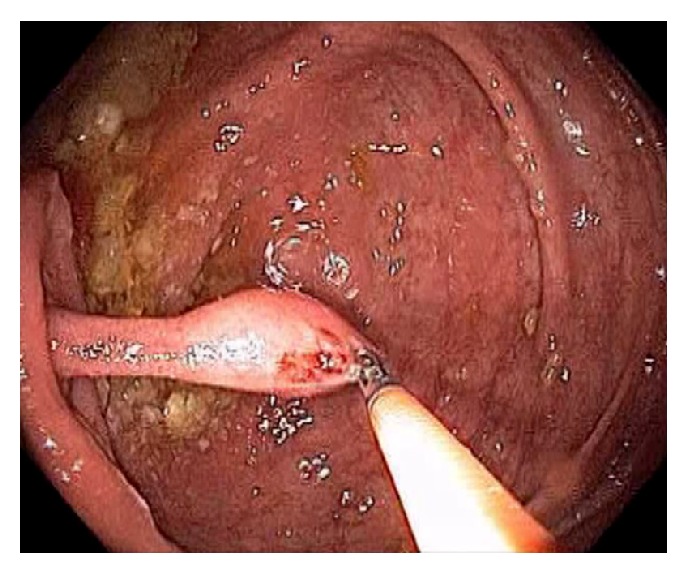
Endoscopic image of a pedunculated intraluminal lipoma arising from the terminal ileum.

Neoplasms of the TI can present with abdominal pain, GI bleeding, and obstructive symptoms. On endoscopy, they may appear as nodularities, induration with erythema, erosions, and ulcerations. They can also appear as polypoid or pedunculated lesions causing prolapse or intussusception. Malignant neuroendocrine neoplasms of the TI can be locally invasive and metastasize to regional lymph nodes or liver. Occasionally, we encounter neoplasms of the TI that can be removed endoscopically (Figure 34).

**Figure 35:**
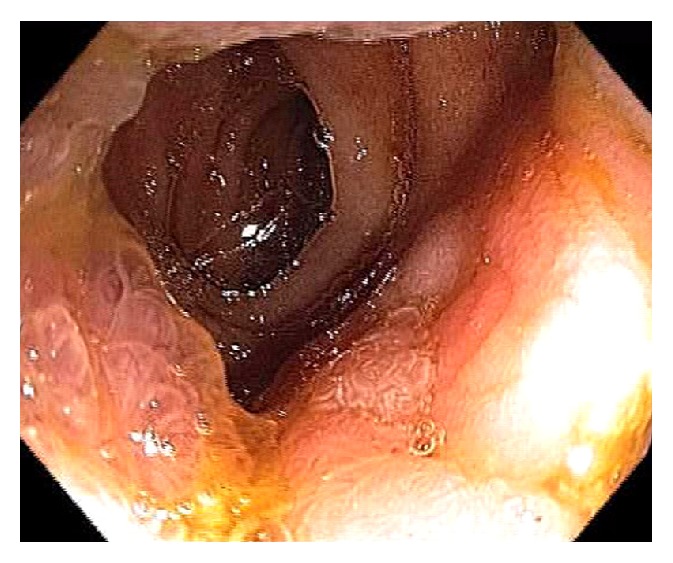
Endoscopic image of a large sessile ileocecal valve adenoma extending into the terminal ileum.

During the examination of the IC valve, the endoscopist needs to consider the possibilities of small bowel mucosal prolapse or metaplasia, or lipomatous IC valve. In general, the adenoma of the ileocecal valve is sessile. Endoscopic mucosal resection of laterally spreading adenomas involving the IC valve can be challenging. Recently, a large single center study showed complete adenoma clearance rate of 93.6* *% and surgery was avoided in 81.1* *% [81]. These patients need surveillance colonoscopy for the detection and treatment of possible local recurrence. Factors associated with failure of endotherapy were adenoma TI extension and involvement of both IC valve lips [80] (Figure 35).

**Figure 36:**
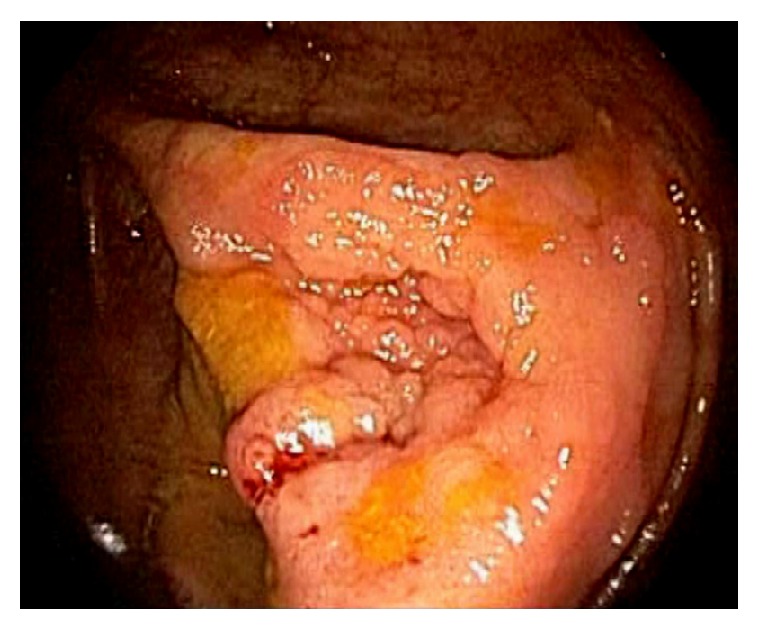
Endoscopic image of a large cecal polyp involving the appendiceal orifice and almost the entire cecal pole.

Cecal adenomas are generally sessile in morphology. In order to remove a large polyp, submucosal injection should be performed. Piecemeal polypectomy or en bloc submucosal dissection of large cecal polyps is now feasible and considered relatively safe. When the cecal adenoma is extensive or wide spread, with limited endoscopic skills, surgical resection should be considered (Figure 36).

**Figure 37:**
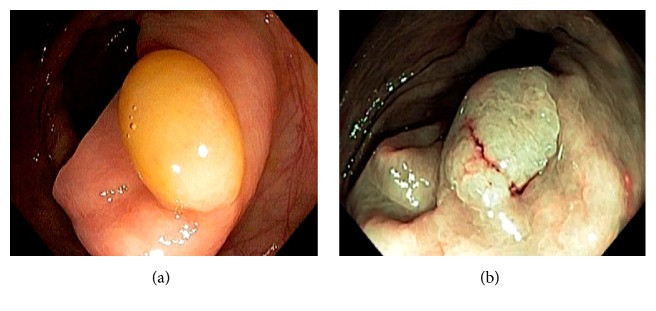
Endoscopic image of a serrated sessile adenoma: the adenoma is covered by a mucus cap or mucoid covering (a); after the mucus cap is washed off, the adenoma is examined under digital chromoendoscopy (b).

Increasingly, sessile serrated adenomas and pathway are being recognized to play an important role in the development of right-sided colon cancer [82, 83]. Frequently, mucus cap or mucoid covering can be seen endoscopically (Figure 37). They are generally flat, sessile, or slightly polypoid. They can mimic a mucus collection or a lipoma. The mucus cap can be easily washed off, exposing the underlying serrated adenoma or polyp. The presence and the extent of the lesion can be highlighted by digital chromoendoscopy and by submucosal injection of a contrast agent. In a recent study, the most site to harbor an sessile serrated adenoma-high-grade dysplasia is the cecum and proximal ascending colon [83].

**Figure 38:**
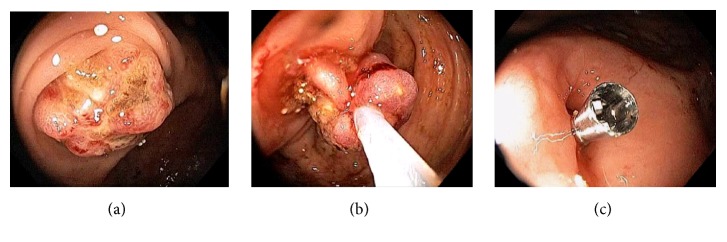
Endoscopic image of a 12 mm appendiceal adenoma with high-grade dysplastic features (a). On gentle manipulation of the ensnared lesion, the polyp is found to be pedunculated (b). In order to position the snare at the polyp stalk, the endoscopic catheter is gently advanced into the orifice before closing the snare. In order to achieve hemostasis for mild oozing, a semiopened endoclip is advanced into the orifice and applied at the base (c). On pathological examination of the resected polyp, there was focal high-grade dysplasia but the resection was considered curative.

For diminutive adenoma or polyp at the appendiceal orifice, endoscopic excisional biopsy can be performed. For small adenoma, the authors recommend submucosal injection followed by polypectomy, if the lesion does not extend into the appendiceal orifice and can be completely removed. In the authors' experience, unlike other locations in the colon, the mucosal lifting is limited due to the evagination of the appendix. For pedunculated polyp arising from the appendix, endoscopic removal can be attempted with or without preinjection (Figure 38). Endoscopic loop or clip application can be considered to minimize the risks of bleeding [84]. If the polyp is noticed to be extending into the appendiceal orifice, with current endoscopic methods and device, complete endoscopic removal is not feasible. The patient should be referred for elective surgical resection.

**Figure 39:**
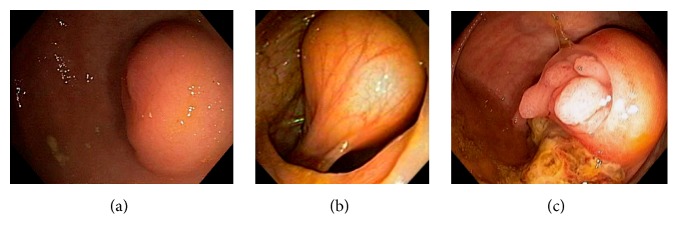
Endoscopic images of appendiceal mucoceles. The first mucocele is caused by a retention cyst or mucosal hyperplasia (a). The second mucocele is caused by a mucinous cystadenoma (b). A mucinous adenocarcinoma is responsible for the third mucocele (c). A large amount of mucin is present in the cecum.

**Figure 40:**
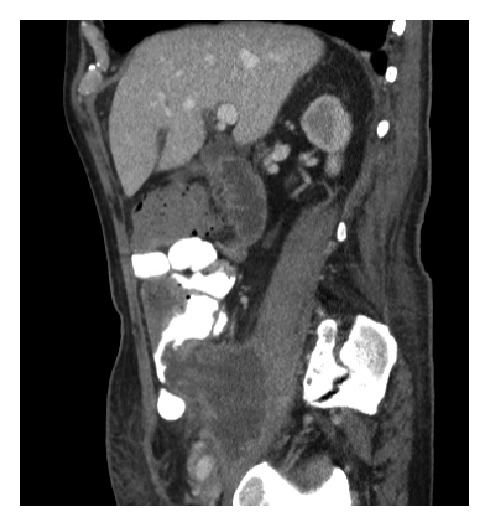
Computer tomographic image showing a ruptured mucocele from mucinous adenocarcinoma leading to pseudomyxoma peritonei.

A mucocele represents the accumulation of mucoid material within the appendix. It can be a simple retention cyst, mucosal hyperplasia, or as a result of neoplastic process, such as mucinous cystadenoma and cystadenocarcinoma [85, 86] (Figure 39). The reported incidence is 0.2% to 0.3% of appendectomy specimens [85, 86]. It can be an incidental endoscopic finding or cause right lower abdominal pain, nausea, vomiting, chronic GI bleeding, acute appendicitis, appendiceal mass, perforated appendix, intussusception, and pseudomyxoma peritonei. On endoscopy, mucocele appears as a submucosal bulge or nodular lesion at the cecal pole around the appendiceal orifice. Occasionally, mucinous discharge can be observed emitting from the appendiceal opening. A combination of colonoscopy and CT scan can be used to evaluate suspected cases (Figure 40). All patients with a mucocele deserve a surgical consultation.

**Figure 41:**
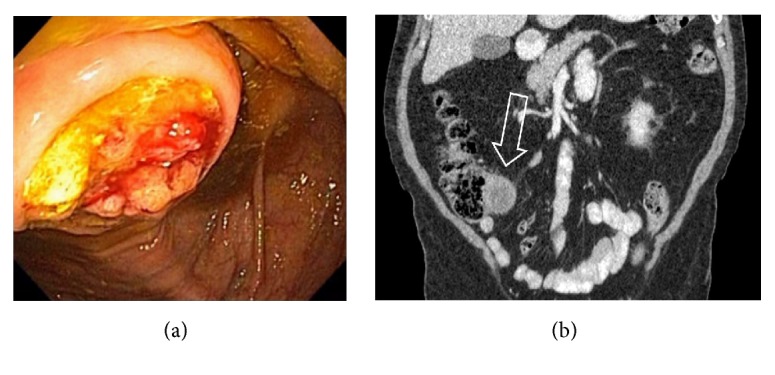
Endoscopic image of a primary appendiceal mucinous adenocarcinoma (a). A 6 cm appendiceal mass (arrow) is seen on computer tomography (b).

**Figure 42:**
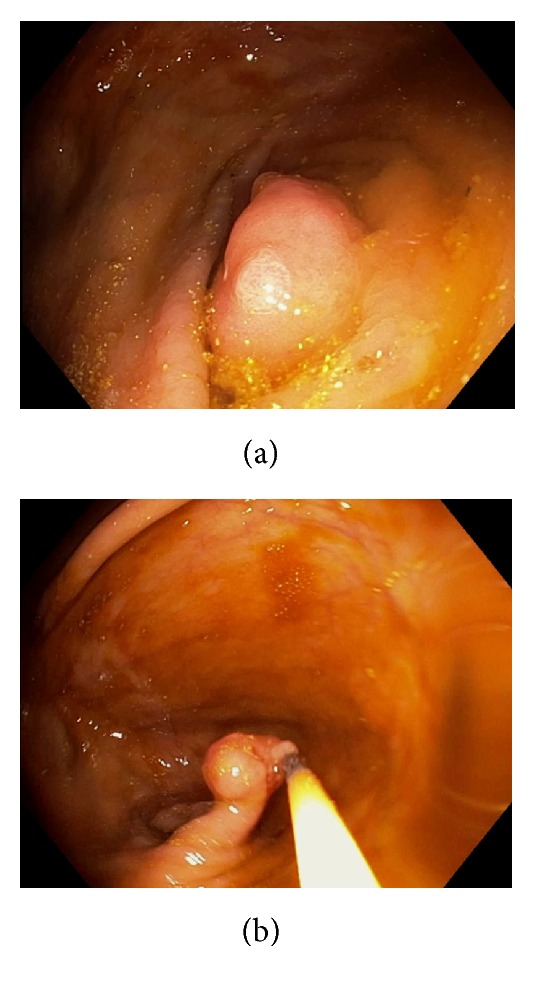
Endoscopic images of an appendiceal submucosal neuroma with a diminutive tubular adenoma on the top (a). The neuroma is found to be pedunculated and arises from Appendix (b). The lesion was completed removed by snare polypectomy.

Other primary appendiceal malignancies include mucinous adenocarcinoma, carcinoid, goblet and signet-ring cell cancers (Figure 41). The most common appendiceal tumors were mucinous. Benign neoplasms include leiomyoma, lipoma, and neuroma (Figure 42). In recent comprehensive reviews, carcinoids presented at an earlier mean age of 41 years and 71% were female [87, 88]. Current guidelines recommend that a right hemicolectomy be performed for all carcinoid tumors >2 cm. Overall five-year survival was highest for carcinoid (83%) and lowest for signet ring (18%).

**Figure 43:**
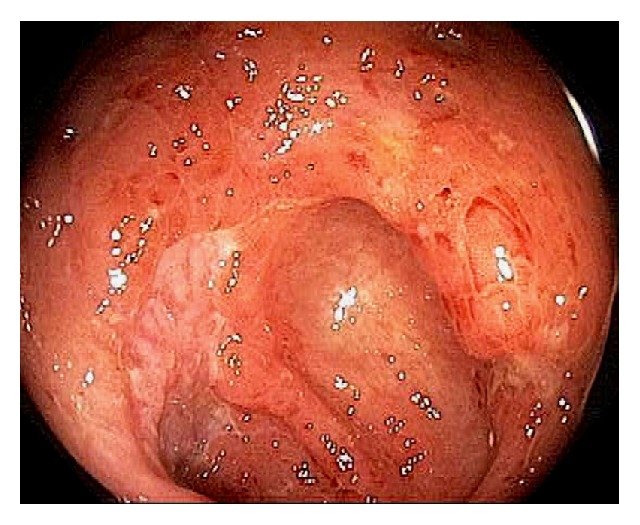
Endoscopic image of the ileocecum involved by B-cell lymphoma.

**Figure 44:**
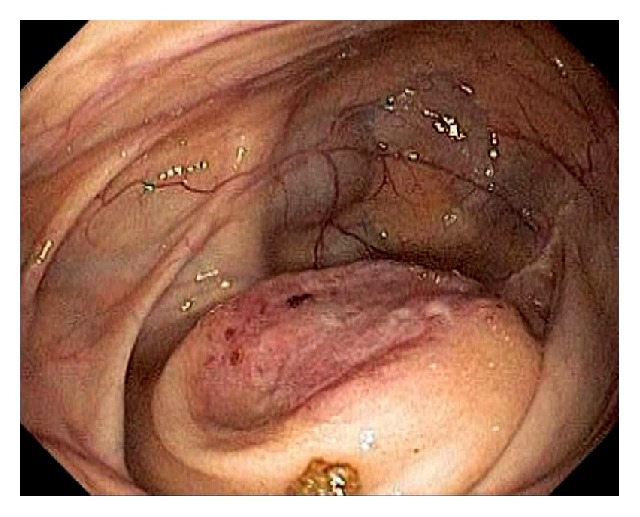
Endoscopic image of a secondary appendiceal cancer from a primary lung neoplasm.

The GI tract is the most common extranodal site involved by lymphoma with the majority being non-Hodgkin type [89]. The most frequent sites are the stomach, small bowel, and ileocecum (Figure 43). Diffuse large B-cell lymphoma is the most common pathological type of GI lymphoma. Certain neoplasms have higher tendency to metastasize to the intestines, including melanoma, lung and breast cancer (Figure 44). Additionally, bowel endometriosis affects between 3.8% and 37% of women. IC endometriosis can mimic Crohn's disease, mass with severe mucosal edema, ileocolic intussusception, and luminal stricture in the distal ileum [90, 91]. In patients with infiltrating rectovaginal and IC endometriosis, bowel resection is required in reducing pelvic pain, constipation, and dyschezia [92].

**Figure 45:**
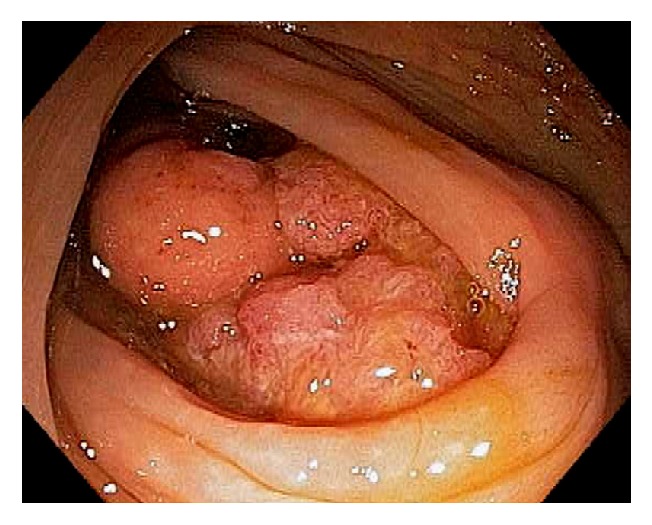
Endoscopic image of a missed cecal cancer after a recent “normal” diagnostic colonoscopy for iron deficiency anemia.

Before the advent of CT scan and ultrasound, radiological imaging the appendix was achieved through contrast filling of the appendix (appendiculography) [57]. Not in the remote past, colonoscopic appendicography is described with contrast injection into the appendix during endoscopy [58]. More recently, direct endoscopic imaging within the appendix with through-the-channel mini scope was also reported [59]. With continuing technological advancement and evolution, diagnostic and therapeutic “appendicoscopy”, endoscopic ultrasound assessment of the ileocecum and appendix, endoscopic transmural resection of neoplasm and appendix, in vivo optical diagnosis, and evaluation of early dysplasia within ileocecum will become a reality. To conclude this review, the authors would like to stress three principles during endoscopic examination of the ileocecum: optimal colon preparation, recognition and photodocumentation of the ileocecal landmarks, and systematic and careful examination of this region [93]. The aims are to diagnose early dysplastic lesions and to reduce the missed cancer and interval cancer rates associated with screening colonoscopy (Figure 45).

## Acronyms

IC: Ileocecal
TI: Terminal ileum
GI: Gastrointestinal
IBD: Inflammatory bowel disease
SMA: Superior mesenteric artery
CT: Computed tomography
UC: Ulcerative colitis.
